# Supplementary material for: The Ameliorative Effect and Mechanisms of Ruditapes philippinarum Bioactive Peptides on Obesity and Hyperlipidemia Induced by a High-Fat Diet in Mice
Source: Nutrients. 2022 Nov 28;14(23):5066. doi: 10.3390/nu14235066 (PMC9737393; doi:10.3390/nu14235066)
Supplement: Supplementary file 1 [file nutrients-14-05066-s001.zip › nutrients-1997258-supplementary.pdf]

Supplementary Table S1. Contents of main components of RBPs

	Crude protein content (%)	Total sugar (%)	Inorganic salt (%)	Moisture content (%)
RBPs	71.10 ± 1.25	19.90 ± 0.58	4.02 ± 0.89	5.00 ± 0.94

All the data presented are the mean ± standard deviation (SD).

Supplementary Table S2. Formulation of high fat feed

Component	Composition ratio of high fat feed (g/100g)
Corn starch	50.00
Lard	16.90
Sucrose	15.00
Casein	10.8
Cholesterol	1.3
Cholate	0.3
Mixed vitamin	2.0
Maltodextrin	3.7
Total	100

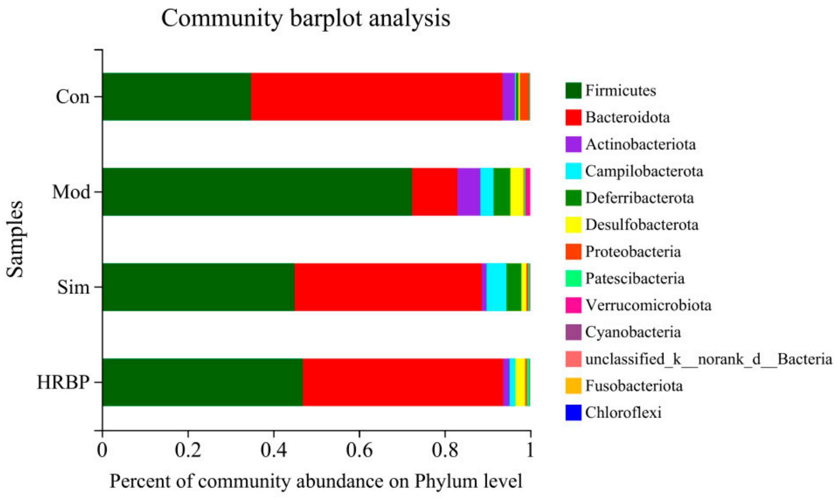

Supplementary Figure S1. Composition of gut microflora at the Phylum level. With the vertical coordinate as the group name, the horizontal coordinate as the proportion of the species in the sample, the columns of different colors represent different species and the length of the columns represents the proportion of the species.
